# Primary breast angiosarcoma: A case report

**DOI:** 10.3389/fsurg.2022.966792

**Published:** 2023-02-17

**Authors:** Yu He, Liyuan Qian, Lang Chen, Yang Liu, Yanguang Wen, Peiguo Cao

**Affiliations:** ^1^ Department of Oncology, Third Xiangya Hospital, Central South University, Changsha, China; ^2^ Department of Breast and Thyroid Surgery, Third Xiangya Hospital, Central South University, Changsha, China; ^3^ Department of Hepatobiliary and Pancreatic Surgery, Third Xiangya Hospital, Central South University, Changsha, China; ^4^ Department of Pathology, Third Xiangya Hospital, Central South University, Changsha, China

**Keywords:** breast angiosarcoma, chemotherapy, radiotherapy, surgery, DSA (digital subtraction angiography), case report

## Abstract

**Background:**

Primary breast angiosarcoma (PBA) is a rare sarcoma, accounting for only 0.04% of all breast malignancies, with a difficult diagnosis and a poor prognosis. Mastectomy is the standard treatment, and the role of adjuvant treatment (chemotherapy and/or radiotherapy following surgery) remains uncertain with very few studies.

**Case Presentation:**

We report the case of a 17-year-old female patient who presented with a right breast lump that had rapidly increased in size and was hemorrhaging. She was diagnosed with breast angiosarcoma by needle biopsy and pathological evaluation. However, the mass showed a quick tendency to bleed during biopsies. After that, we performed angiography and tumor vascular embolization. The patient underwent a mastectomy followed by adjuvant chemotherapy.

**Conclusion:**

Tumor vascular embolization reduced the surgical risk of PBA with hemorrhage complications. Postoperative therapeutic roles still need further exploration and verification.

## Introduction

Breast angiosarcoma is an extremely rare and aggressive malignancy that originates from vascular or lymphatic endothelial cells, accounting for 0.1%–0.2% of all breast neoplasms. It can be divided into primary and secondary angiosarcoma. It has a poor prognosis, with a 5-year survival rate of 40% (1). Primary breast angiosarcoma (PBA) is extremely rare (2). Currently, the most common management approach is surgical excision without involved margins (R0 resection) (3). We report the case of breast angiosarcoma in a patient who appeared with a large lump and bleeding in the right breast.

## Case presentation

**Figure 1:**
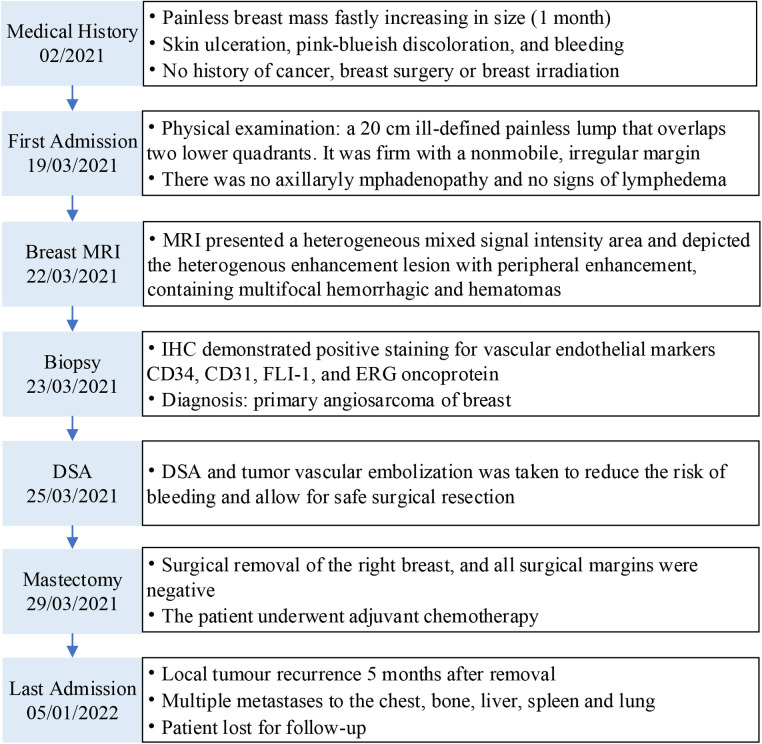
Case report timeline. MRI, magnetic resonance imaging; DSA, digital subtraction angiography; IHC, immunohistochemistry.

In February 2021, a 17-year-old female patient presented with a lump on the right breast about 1.5 × 1.5 cm in size in the lower inner quadrant (Figure 1). The lesion gradually increased in size over a period of 1 month without any pain or bleeding. She did not consult any doctor until the right breast underwent rapid enlargement after accidentally falling, reaching 20 × 20 cm. She had skin ulceration, pink-blueish discoloration, and bleeding from the breast lump for 3 days, without heat, pain, or discharge. On physical examination, the lesion appeared firm with a non-mobile, irregular margin. Axillary lymphadenopathy was negative, and there were no palpable supraclavicular nodes. The left breast was normal.

There was no other remarkable feature in the patient's medical history. She had no history of chronic illness or radiotherapy and was without a significant medical family history, including breast cancer. During hospitalization, the results of the laboratory tests were as follows: WBCs, 7,760/mm^3^ (normal: 3,500–9,500/mm^3^), Hb, 5.1 g/dL (11.5–15.0 g/dL), hematocrit, 18.0% (35.0%–45.0%), platelet, 80,000/mm^3^ (125,000–350,000/mm^3^), PT, 17.9 s (9.0–14.0 s), APTT, 39.8 s (20.0–40.0 s), INR, 1.57 (0.8–1.5), fibrinogen, 60 (200–400) mg/dl, D-dimer, 85.27 mg/L (0–0.55 mg/L). An ultrasound examination was performed, demonstrating that the right breast had heterogeneously mixed echogenicity of approximately 254 × 280 × 96 mm with an indistinct margin.

**Figure 2:**
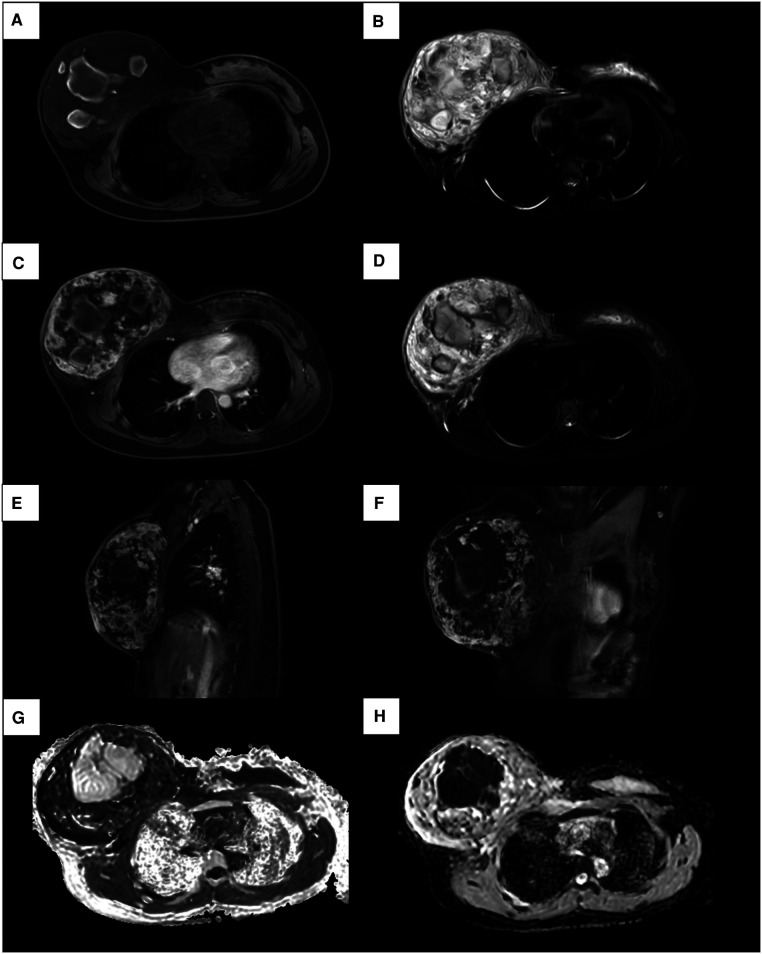
MRI images of a 17-year-old female patient with right primary breast angiosarcoma. (**A**) MRI images show a mixed signal intensity area predominantly composed of isointense mass on T1. (**B**) Mixed signal intensity area predominantly of high signal intensity on T2. (**C, E, F**) Enhancement sequences image showed irregular heterogeneous high signal intensity. (**D**) On fat-suppressed T2-weighted images, the lesion shows bright (high) signal intensity. (**G**) High signal intensity on DWI and (**H**) low signal intensity on ADC images demonstrate restricted diffusion.

Considering the inconclusive findings on ultrasound, computed tomography (CT) scans of the head and chest were performed and revealed that the right breast had heterogeneously increased in density, with focal skin thickening, and an enhancement of the nipple. There was no obvious axillary lymphadenopathy in both axillae. In addition, the magnetic resonance imaging (MRI) showed a heterogeneous mixed signal intensity area predominantly of isointense signal intensity on T1-weighted images, and high signal intensity on T2-weighted images, containing multifocal hemorrhages and hematomas. Subsequent contrast-enhanced MRI depicted the heterogenous enhancement with peripheral enhancement (Figure 2). An ultrasound of the abdomen and an F-18 bone scan were negative for metastatic disease.

**Figure 3:**
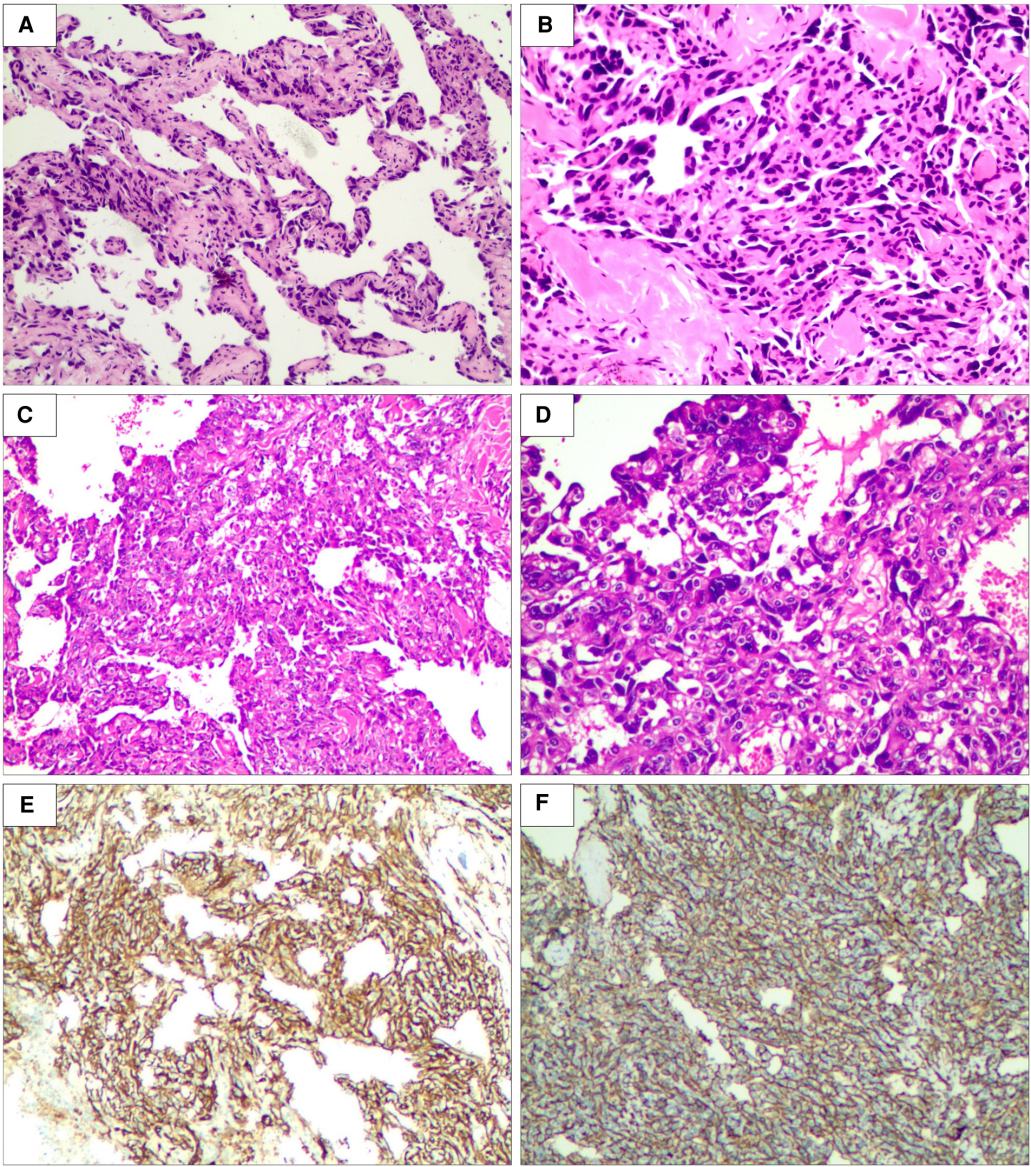
Biopsy of right breast lesion microphotography. The tumor is composed of atypical endothelial cells with different degrees of differentiation, forming irregular vascular cavities that coincide with each other, forming papillary and diffuse infiltration (×200, (**A**) punch biopsy and (**C**) excisional biopsy). Tumor cells exhibit fusiform or irregular shapes with little cytoplasm and hyperchromatic nuclei (×400, (**B**) punch biopsy and (**D**) excisional biopsy). Special stain CD31 positive (**E**). Special stain CD34 positive (**F**) (hematein–eosin staining).

Thereafter, a right breast ultrasound-guided needle biopsy was performed. Vascular channels were lined by atypical, plump endothelial cells with hemorrhage and focal mitotic activity. Morphology suggested the possibility of angiosarcoma. Further immunohistochemistry demonstrated positive staining for vascular endothelial markers CD34, CD31, FLI-1, and erythroblast transformation-specific (ETS)-related gene (ERG) oncoprotein but not for cytokeratin (Figures 3A, B, E, F). The proliferation index (Ki-67) was estimated at 40%. So, a diagnosis of primary angiosarcoma of the breast (pT4N0M0) was made.

**Figure 4:**
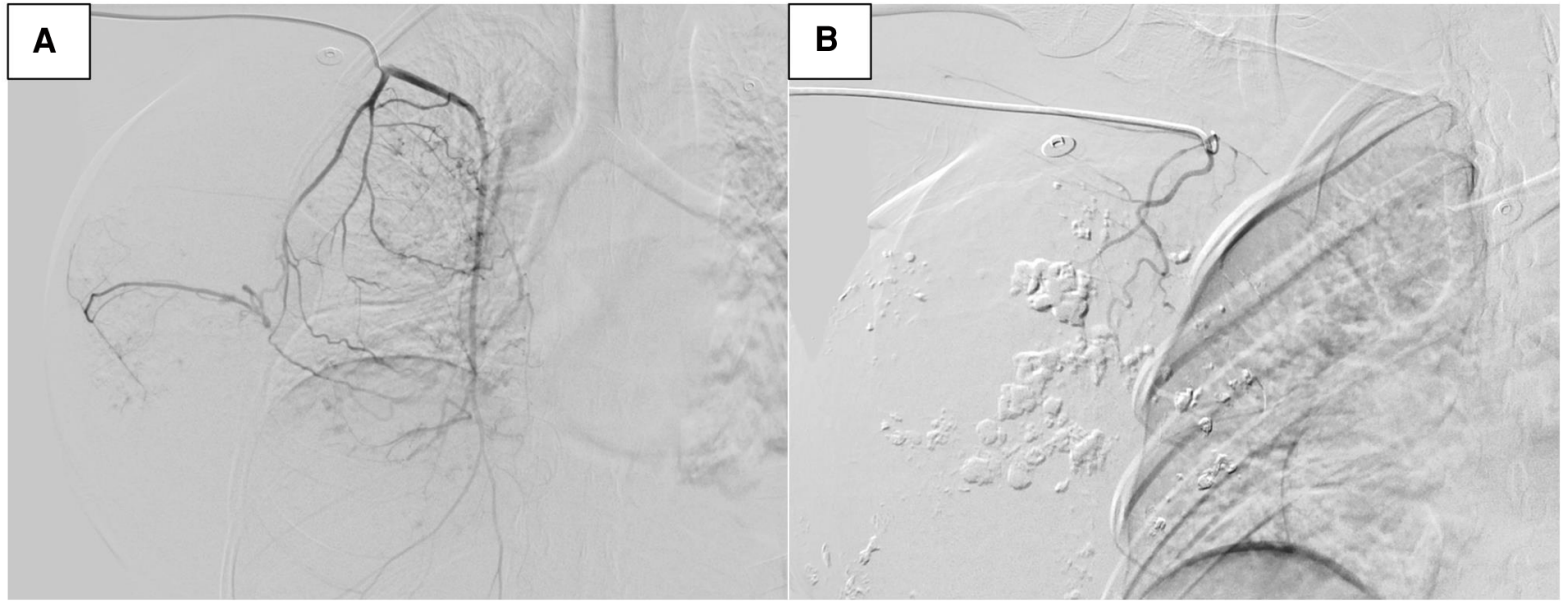
Digital subtraction angiography (DSA) image of tumor vascularization before embolization (**A**). DSA after embolization (**B**).

The patient had coagulation dysfunction for tumor-related excessive bleeding, decreased fibrinogen and platelet count, prolonged prothrombin time, and elevated D-dimer levels. She received red cell concentrates, cryoprecipitate, fibrinogen, platelets, and fresh-frozen plasma, but the anemia and coagulation did not improve significantly. A decision to undertake angiography (DSA) and tumor vascular embolization was taken to reduce the risk of bleeding and allow safe surgical resection (Figure 4).

**Figure 5:**
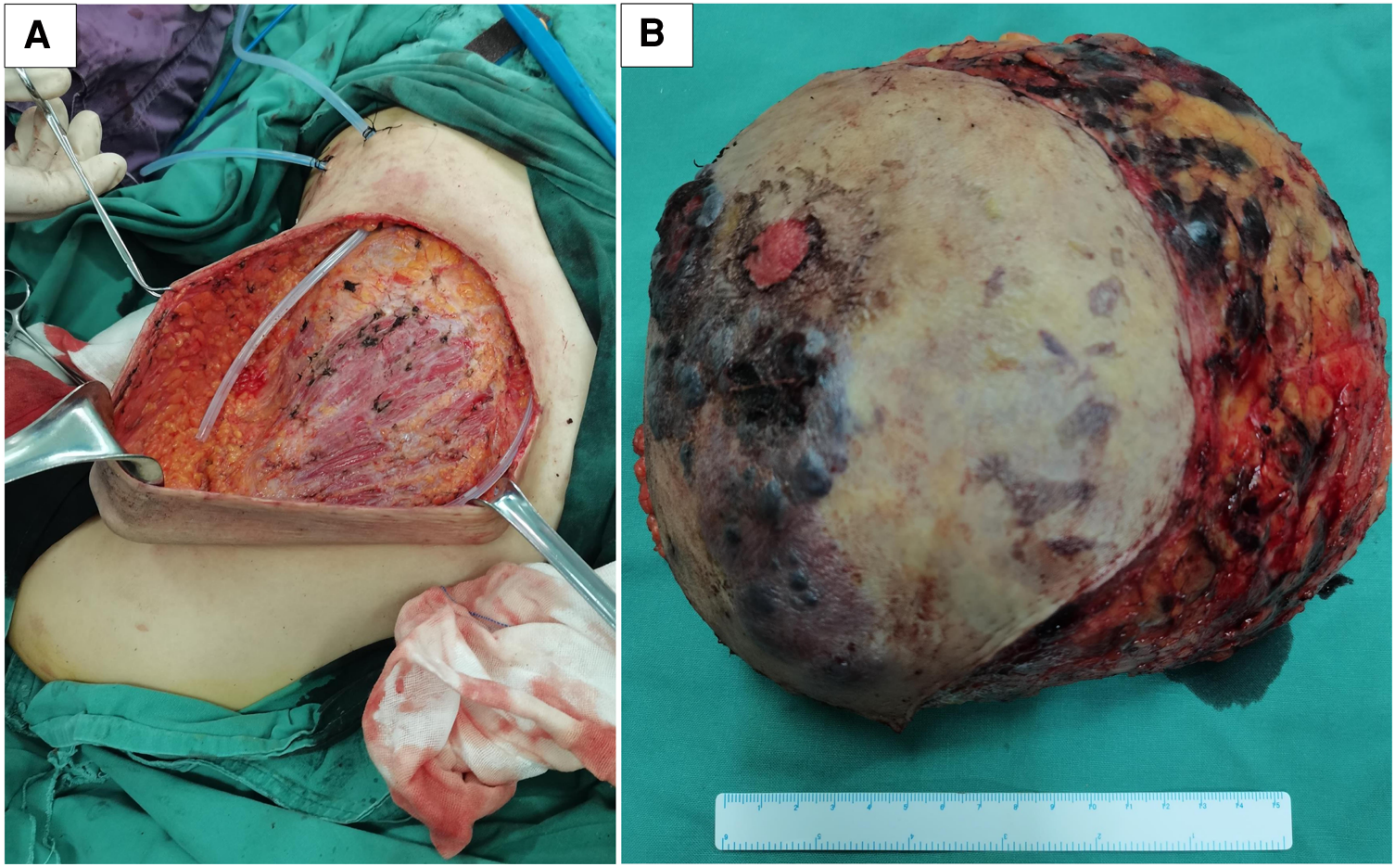
The patient was operated on successfully with less amount of blood loss (**A**). In the mastectomy specimen section of the right breast, the tumor was ill-defined, with a focal area of necrosis and hemorrhage (**B**).

After the risks associated with the surgery were explained to the patient and her family, she accepted them and wanted to be operated on as soon as possible. She underwent a right total mastectomy in March 2021, and all surgical margins were negative. Postoperative pathological examination revealed angiosarcoma with tissue necrosis, hemorrhage and 19.5 × 15.3 × 6.6 cm in size (Figure 5). Histopathologic examination showed the tumor was composed of atypical endothelial cells with different degrees of differentiation, forming irregular vascular cavities that coincide with each other, forming papillary and diffuse infiltration. Tumor cells exhibit fusiform or irregular shapes with little cytoplasm and hyperchromatic nuclei (Figures 3C, D).

After consultation with the patient and her family, she received planned radiotherapy and chemotherapy. The patient received Adriamycin-based chemotherapy every 3 weeks for eight cycles. Medical oncology planned a 50 Gy intensity-modulated radiation therapy to the chest wall (2 Gy per fraction over 5 weeks), but she was unable to complete the radiation therapy due to her poor physical condition.

The patient presented with a 3 cm × 1.5 cm bleeding right chest wall mass 5 months after removal. She was treated with surgical resection with a diagnosis of local tumor recurrence by histological examination. She presented again with a chest wall tumor that was bleeding profusely 1 month after surgery for a recurrent tumor and was admitted to the emergency department. A chest and abdominal CT scan with contrast showed multiple metastatic lesions in the chest, bone, liver, spleen, and lung. She was anemic despite the transfusion. The patient's condition has not yet improved. She left the hospital arbitrarily.

## Discussion

Angiosarcoma is an extremely rare and highly malignant mesenchymal vasoformative neoplasm, characterized by rapidly proliferating and extensively infiltrating growth (4), most commonly identified in the skin of the head, neck, and scalp; the breast is an exceedingly rare primary site of occurrence (5). It occurs especially in women 30–40 years of age. The median survival time and 5-year recurrence-free survival rate are 24 months (about 2 years) and 33%, respectively (6). Currently, for the PBA, the underlying cause remains unknown. It represents about 0.05% of all malignant breast tumors (7). Primary lesions affect younger patients, and the median age is 30–50 years (8). Patients usually present with an associated palpable mass in the breast. The prognosis of patients diagnosed with PBA depends on the grade of the tumor. In patients with grade 1 tumors, the corresponding 5-year disease-free survival after the initial treatment is approximately 76%, whereas the probability drops to 15% for grade 3 tumors (9). In comparison, secondary breast angiosarcoma (SBA) is identified as chronic lymphedema resulting from axillary dissection (Stewart-Treves syndrome) or radiation-associated sarcoma (10). Radiotherapy used to treat invasive breast tumors is a well-known risk factor for the development of the so-called radiation-induced angiosarcoma (RIAS). RIAS is a late toxicity that occurs in 0.05%–0.3% of breast cancer patients who undergo breast-conserving surgery and adjuvant radiotherapy. It usually occurs 6–10 years after breast irradiation (11), but RIAS can occur as early as 1–2 years or as late as 41 years after radiation (12). SBA is relatively common in older women, as the median age is 70 years (13). About 5-year survival rates remain low, at 22.5% for secondary angiosarcoma (7). Because the clinical and radiologic findings are not specific (14), the definitive diagnosis can only be achieved by histopathological examination.

PBA originates from breast parenchyma and occasionally affects the skin (15), while SBA often involves the skin, and rarely the breast parenchyma. PBA has similar histologic and morphologic features to SBA. Tumor size ranges between 10 and 160 mm (mean: 59 mm) for post-radiation angiosarcomas and 25–150 mm (mean: 76 mm) for PBA. The pathology of angiosarcoma shows grade 1 (low grade) angiosarcoma with inter-anastomosing vascular channels, subtle endothelial atypia and relatively few mitotic figures. Grade 2 (intermediate grade) tumor cells show moderate nuclear atypia and multilayering of endothelial cells. Grade 3 (high-grade) tumors present with marked nuclear polymorphism, numerous mitoses, and necrosis (16). Most RIAS are high-grade lesions with irregular anastomosing vessels lined by endothelial cells showing nuclear atypia (17). The tumor cells are usually positive for vascular markers (e.g., CD31, CD34, factor VIII-related antigen, FLI1, Ulex europaeus 1 lectin, and ERG) (18). The histological and pathological features are similar between primary and post-radiation angiosarcomas. Therefore, they could not be differentiated on pathological examination (19). Currently, the pathogenesis of angiosarcoma is not completely known.

Several physiopathological mechanisms have been proposed to explain the development of RIAS. Radiotherapy uses ionizing radiation, which either directly affects DNA structure by inducing DNA strand breaks, particularly double strand breaks, or indirectly by generating reactive oxygen species (ROS) that oxidize proteins and lipids and thereby induce additional damage to DNA, like the generation of abasic sites and strand breaks (20). Genome instability and cancer-related gene mutations may drive tumorigenesis (21).

Although radiation-associated angiosarcoma presents a distinct clinical pattern, the difference between radiation-induced vs. sporadic lesions remain unverified. The establishment of genetic differences between sporadic and radiation-induced angiosarcomas will facilitate discrimination between these two entities (22). As of RIAS, several gene mutations have been reported in the literature. All molecular studies carried out on radiation-induced angiosarcomas present amplification of chromosome 8q24 mapping, Myc oncogene inactivation, and the expression of the p53 gene (23).

Oncogenes of the Myc family, including c-Myc, N-Myc, and L-Myc, are master regulators of cell growth, maturation, and death, because of their transcriptional repression function. The fundamental pathogenetic differences between PBA and RIAS are impossible to differentiate morphologically. Some studies have found that Myc amplification frequently presents in secondary angiosarcoma (radiation, chronic lymphedema) and less frequently in primary angiosarcoma, suggesting that it may be utilized to distinguish RIAS of the breast and PBA (17, 19, 24–26). However, Myc overexpression could also be seen in primary angiosarcoma (27, 28).

The morphological profile of atypical vascular lesions (AVL) showing progression to angiosarcoma has not yet been clearly defined. A study identified some tumor protein p53 (TP53) variation from radiotherapy-induced ipsilateral breast carcinoma in 10 out of 12 (83.3%) cases of AVL and in 7 out of 8 (87.5%) cases of angiosarcoma. At the protein level, the elevated expression of p53 and MDM-2 proteins has been found to associate with the increased vascular endothelial growth factor (VEGF) expression that is found in nearly 80% of angiosarcoma. The genetic alterations of the TP53 gene suggest that its mutational inactivation may be implicated in the pathogenesis of vascular proliferations associated with radiotherapy (29, 30).

The VEGF family and VEGF receptors (VEGFR) that control angiogenesis are found to be frequently altered in angiosarcoma. The genetic mutation VEGFR-2 (kinase insert domain receptor = KDR) or amplification of VEGFR-3 (FMS-like tyrosine kinase 4 = FLT4) have been proposed to have a crucial role in the development of angiosarcoma (31, 32). Mutations in KDR and TP53 were mutually exclusive (*P* = .02, Fisher's exact test), with 8 out of 9 (89%) KDR missense mutations displayed in PBA samples and 9 out of 11 (82%) TP53 missense mutations observed in angiosarcoma samples that were not PBA (33). However, Guo et al. found that the gene amplification of the FLT4 gene encoding the VEGFR3 was found in 25% of secondary angiosarcoma and only associated with Myc amplification. Based on those results, they suggest that FLT4 overexpression may represent a “second hit” in the progression of secondary angiosarcoma and raise a note-worthy possibility of targeting FLT4 as a potential therapeutic option (26). Although activating VEGFR-2 mutations are relatively rare in angiosarcoma, VEGFR-2 is universally overexpressed in angiosarcoma (17, 34, 35). Itakura et al. showed a lower percentage of VEGFR-2 expression was significantly associated with poorer overall survival (31). Uncontrolled VEGF/VEGFR signaling leads to dysregulated angiogenic activity, however, the exact mechanism in angiosarcoma remains to be elucidated.

Previously published case reports have shown that radiation-induced angiosarcoma contained breast cancer-related tumor-suppressor gene BRCA1/BRCA2 mutations (11, 36–38). The defective DNA repair mechanism may also theoretically increase radiosensitivity, increasing susceptibility to carcinogenic effects in surviving cells (39, 40).

The PIK3CA (phosphatidylinositol-4,5-bisphosphate 3-kinase catalytic subunit alpha) gene coding for the p110alpha catalytic subunit of class 1A phosphoinositide-3-kinase (PI3K) is frequently mutated in breast cancer (41). Intriguingly, a study showed that 6 out of the 10 PIK3CA alterations were found in PBA samples (32). In addition, another study showed that 9 out of the 10 PIK3CA alterations were found in PBA samples, whereas PIK3CA mutations were significantly enriched in the angiosarcoma subtype compared with other subtypes (9 out of 18 PBA samples vs. 1 out of 29 angiosarcoma samples that were not PBA; *P* = .0003, Fisher's exact test). DNA methylation may serve as a marker of breast tumor cell lineage restriction, thereby reflecting the cell type from which cancer originates and, perhaps, explaining the correlations between the histological heterogeneity and prognosis of breast cancers with their DNA methylation profiles. Each type of activating PI3K mutation is derived from a different lineage of breast malignancies, indicating the site the tumor had originated independently of tumor lineage. It may play an important permissive role in PI3K pathway activation and may provide an interaction with the breast microenvironment conducive to new tumor formation. Of clinical importance, these findings suggest that targeting PI3Kα *via* inhibitors may be useful as a novel therapeutic intervention for patients with PBA (33).

Wei et al. (42) explored the mechanisms of primary and secondary breast angiosarcoma for the discovery of new biomarkers and research into potential therapeutic targets. The study identified 18 differentially expressed genes (DEGs) enriched in the transforming growth factor-β (TGF-β), Wnt, Hippo, and PI3K-Akt signaling pathways. It is possible that genomic testing will help differentiate between the two clinical entities and lay the foundation for the discovery of effective and reliable molecular biomarkers and essential therapeutic targets in the future.

In addition, several other genetic alterations, including POT1, RAS, BRAF, PTPRB, PLCG1, ATM, MSH6, and APC, may be associated with angiosarcoma, although further research is required (43, 44). Table 1 includes clinical, demographic, and genetic variation data for SBA and PBA.

**Table 1:** Summary of clinical features and genetic variations in various series of patients with breast angiosarcoma.

Author	AS	Age Dx (years)	Number of cases	AS location	BC Stage	ER/PR/Her2	BC Rx	Genetic variations	RT dose (Gy)	Time from RT to AS (years)	AS Rx	F/U (years)
Retter et al. (24)	SBA	67	1	Lt	T1aN0M0	−/+/−	LUM + L + H	c-MYC	50.4 + 12.5	10	BS	Alive NED (2)
Vin Chang et al. (45)	SBA	80	1	Rt + Lt	T1cN0M0	+/+/−	BS + L + C + H	KRAS, PIK3CA, RPTOR, VHL, MYC	Yes	8	BS + RT + C	Dead MT (1)
Sheu et al. (46)	SBA	79	1	Rt	TxNxM0	+/−/ND	BCS + AND + C + H	MYC, NOTCH1	Yes	14	TM	ND
Oliveira et al. (47)	SBA	73	1	Rt	T2N1M0	+/+/−	BCS + AND + C + H	c-MYC	50 + 10	6	SM	Alive NED (2)
Webb et al. (48)	SBA	34	1	Rt	ND	ND	BS	BRCA1, c-MYC	No	10	BS	ND
Shiraki et al. (49)	SBA	72	1	Rt	T1bN0M0	+/+/−	BCS + H	c-MYC	50	5	M + C	Dead MT (2.6)
Manjee et al. (50)	SBA	70	1	Lt	ND	ND	LUM	MYC	Yes	7	M	ND
Mentzel et al. (51)	SBA	36–83	20	Br				c-MYC	Yes			
Daniels et al. (52)	SBA	71 (48–94)	10					MYC	Yes	6.5 (4–14)		
Tidwell et al. (53)	SBA	68	1	Rt	TxN0M0	+/+/ND	BCS + AND	c-MYC	34	9	TM	Alive LR (0.25)
Parvez et al. (37)	SBA	62	1	Rt	Lt T1cN0M0 Rt T1bN0M0	+/ND/ND	Lt LUM + AND, Rt LUM + L + C + H	BRCA2	42.5	0.5	BS + RT	ND
Cornejo et al. (54)	SBA	73 (51–91)	17				BCS	13 (76%) MYC, 3 (18%) FLT4, 13 (76%) FLT4	Yes	7.2 (4.3–11.6)		
Silva et al. (55)	SBA	78	1	Lt	ND	ND	BCS + AND	VEGFR2	Yes	11	M + RT + C + TKI	Alive NED (3.4)
Barbosa et al. (56)	SBA	32	1	Rt	T2N0M0	+/+/+	BCS + L + C + H + Trastuzumab	TP53	Yes	3.5	TM	ND
Tajima et al. (57)	SBA	73	1	Lt	ND	ND	C	c-MYC	50	7	TM + C	Alive NED (4.5)
Azzariti et al. (58)	SBA	71	1	Lt	ND	ND	BCS + AND	VEGF	50	9	Lt M + ECT + C + TKI	Alive MT (3)
Fernandez et al. (59)	SBA	74 (58–87)	6					6 (100%) MYC	Yes	7.2 (4–10)	ND	ND
Mentzel et al. (60)	SBA	(46–95)	25					25 (100%) MYC, 24 (96%) MYC, 20 (80%) prox-1	Yes	1.5–13	ND	ND
Teruyama et al. (61)	PBA	52	1					FGFR4, KDR, TP53	No		Rt LUM+C	Dead MT (22)
Shiraki et al. (49)	PBA	80	1	Lt	T2N0M0	+/+/−	BCS + H	c-MYC	No		Lt M + C	Alive LR (1.5)
Al-Salam et al. (62)	PBA	29	1					HIF1α, VEGF, VEGFR, WT-1	50.4		BCS + AND + ANS + Rt + C + avastin + TKI	Alive MT (0.5)
Laé et al. (19)	PBA	36 (19–54)	15					c-MYC 1/15			M + C	5.9 (0.5–23.9)
	SBA	70 (42–89)	32					c-MYC 32/32	50 (32.5–66)	8.8 (3.3–23.2)		3.2 (0.1–9.3)
Fraga-Guedes et al. (63)	PBA	49 (30–77)	12					c-MYC 0/12			ND	9.1 (0.1–13.6)
	SBA	69 (37–88)	37					c-MYC 20/37	Yes	9.1 (1.5–27.6)	ND	9.1 (0.1–13.6)

Dx, diagnosis; BC, breast carcinoma; BC Rx, breast carcinoma treatment; ER, estrogen receptors; PR, progesterone receptors; HER2, human epidermal growth factor receptor 2; ND, non-determined; RT, radiotherapy; C, chemotherapy; H, hormone therapy; TKI, Multi-target tyrosine kinase inhibitors (sunitinib or pazopanib); ECT, electrochemotherapy; BS, bilateral surgery; L, sentinel lymph or lymphadenectomy; AS, angiosarcoma; F/U, follow up; Br, breast; SM, simple mastectomy; TM, total mastectomy; AS Rx, angiosarcoma treatment; M, mastectomy; ANS, axillary node sampling; AND, axillary node dissection; Rt, right; Lt, left; BCS, breast-conserving surgery; LUM, lumpectomy; IHC, Immunohistochemistry; D, dead; NED, no evidence of disease (AS); LR, local recurrence; MT, metastasis.

Our patient, who presented with PBA, is in agreement with a literature review involving younger premenopausal females with no previous history of cancer. The tumor was characterized by rapid growth and bleeding. It was persistent bleeding and hemostasis was difficult to achieve. Ongoing bleeding led to not only coagulation factors, and fibrinogen consumption but also coagulation disorders. Nevertheless, the source of the bleeding in this case could not be identified due to an unclear diagnosis. Furthermore, the clinical and radiologic findings were not specific.

It is frequently difficult to accurately diagnosis a breast lesion as breast angiosarcoma. Differential diagnosis should be made considering both radiological, and histopathology, and immunohistochemistry findings. Differential diagnosis includes benign hemangioma, stromal sarcoma, cystosarcoma phyllodes, metaplastic carcinoma, reactive spindle cell proliferative lesions, fibrosarcoma, myoepithelioma, fibromatosis, liposarcoma, and squamous cell carcinoma with sarcomatoid features (64). This case was initially accepted as a hemangioma. The puncture may result in tumor rupture or great vessel injury, causing hemorrhage and shock. Although a needle biopsy might further aggravate the bleeding risk and can sometimes fail to give definite results, a biopsy is considered the gold standard and an indispensable means of confirming a diagnosis. We performed a biopsy, histological, and immunohistochemical analysis, which revealed the diagnosis of PBA.

Abnormalities in coagulation function make surgery difficult due to the substantial risk of bleeding. Therefore, DSA and tumor vascular embolization were used to reduce the risk of bleeding and allow for safe surgical resection. This treatment successfully stopped tumor bleeding. The assurance of gross tumor resection with tumor-negative resection margins is the primary and preferred treatment modality for localized disease. Lymphatic metastasis is relatively rare, and therefore, axillary node dissection remains controversial in the absence of positive nodes (65). Chemotherapy may help to achieve an improved prognosis in disease control and survival and further reduce the local recurrence rate (9), but the role remains controversial at present (7). A recent study has shown that cytotoxic chemotherapies, in particular anthracycline-based regimens and taxanes can produce significant responses to therapy in a subset of patients (66). Adjuvant radiotherapy could be used to reduce the incidence of locoregional recurrence (67). However, the disease is usually resistant to currently available chemotherapy and radiotherapy, which may not alter the poor prognosis. Sher et al. reported that there was no significant survival difference between patients who had and had not received anthracyclines, taxanes, gemcitabine, and ifosfamide as adjuvant chemotherapy (68). Due to the high-level expression of VEGF in angiosarcoma, therapies targeting VEGF are hopeful to improve prognosis, however, this issue warrants being proven in a properly designed prospective study (69).

Breast angiosarcoma shows hematogenous spread like other sarcomas rather than *via* lymphogenous route. The most common site of recurrence is local-regional. Distant metastases are frequently observed at an early stage. Breast angiosarcoma has been reported to metastasize usually to the liver, bone, lung, skin, central nervous system, spleen, and subcutaneous soft tissues (65, 68). Unlike other sarcomas and breast cancer, lymph node metastasis is exceedingly rare (70). In this case, chest, bone, liver, spleen, and lung metastases were observed.

Given the rarity of PBA, especially with severe bleeding, and the poor prognosis following the identification, knowledge of this lesion prompts further assessment for diagnosis, therapy, and prognosis is crucial when evaluating a patient. The objective and unified criteria for the diagnosis, staging and treatment are still lacking.

## Conclusion

We herein present a case of PBA with severe bleeding in a 17-year-old woman. The definitive diagnosis of PBA is usually difficult, and histopathological examination is the standard approach at present. Mastectomy remains the most favored therapy. Tumor vascular embolization can reduce the risk of bleeding. The role and importance of chemotherapy and radiotherapy in the treatment remains unclear. Furthermore, genetics and genomics will remain powerful approaches to understanding and treating diseases. Therapies targeting VEGFR are hopeful for improving prognosis, but the therapeutic effect still needs further exploration and verification.

## Data Availability

The original contributions presented in the study are included in the article/Supplementary Material, further inquiries can be directed to the corresponding author/s.
